# Case report: Recurrent nocturnal awakenings in cluster headache: a different type of ghost attack

**DOI:** 10.3389/fneur.2023.1230710

**Published:** 2023-07-26

**Authors:** Giada Giuliani, Maurizio Gorgoni, Marta Altieri, Vittorio Di Piero

**Affiliations:** ^1^Department of Human Neurosciences, Sapienza University of Rome, Rome, Italy; ^2^Department of Psychology, Sapienza University of Rome, Rome, Italy; ^3^Body and Action Lab, IRCCS Fondazione Santa Lucia, Rome, Italy; ^4^University Consortium for Adaptive Disorders and Head Pain (UCADH), Pavia, Italy

**Keywords:** cluster headache, nocturnal awakenings, ghost attacks, prophylactic treatment, verapamil

## Abstract

**Introduction:**

Cluster headache (CH) is a trigeminal autonomic cephalalgia characterized by attacks of severe unilateral pain associated with ipsilateral autonomic symptoms. Cluster headache attacks exhibit nocturnal predilection, and sleep disorders could be the first manifestation of an incipient cluster period. Sleep alterations in cluster headache patients may reflect the pivotal role of the hypothalamus, which is crucially involved in the pathophysiology of this primary headache. We describe the case of a patient affected by episodic cluster headache who experienced a sleep disorder after starting therapy with verapamil.

**Case presentation:**

A 47-year-old man was affected by episodic cluster headache, characterized by attacks of excruciating pain in the left orbital and temporal regions, associated with prominent ipsilateral vegetative symptoms. Headaches occurred during the night, with one or two nocturnal attacks appearing at 11.30–12 p.m. and 4–4.30 a.m. Preventive treatment with verapamil was started, with immediate pain relief. Later, he experienced consecutive nocturnal awakenings for a couple of weeks, always at the same time, without any pain or autonomic symptoms. He was not agitated and did not need to get out of bed; after the awakenings, he reported sleep disturbances with vivid dreams.

**Discussion and conclusion:**

This case represents the first description of recurrent cyclic nocturnal awakenings, without pain and autonomic symptoms, in a patient with episodic cluster headache during the active phase of a cluster bout. Nocturnal awakenings, started after the introduction of effective preventive therapy, might be an unusual form of “ghost attacks.” After the beginning of prophylactic therapy, patients often describe mild pain or localized pressure in the same localization of CH attack. Similarly, the appearance of sleep disturbances, without any pain or vegetative symptoms, should be regarded as a warning sign of a still active cluster bout. Since these manifestations may influence therapeutic management, they should be carefully investigated.

## Introduction

Cluster headache (CH) is a trigeminal autonomic cephalalgia characterized by attacks of excruciating unilateral pain, usually associated with prominent ipsilateral cranial autonomic symptoms (1). Painful episodes tend to have a circadian pattern with a nocturnal preference, and their frequency ranges between one attack every other day and eight attacks a day, while the bouts often have a circannual pattern. Patients often describe attacks that wake them ~60–90 min after sleep onset, and they can lucidly recall dreams (2). Because of that, the connection between CH and sleep (especially rapid eye movement sleep) has been extensively studied. The most vulnerable phases for the occurrence of CH attacks seem to be the sleep stage transitions (3). Poor sleep quality and increased sleep latency can be seen even 1 year after the last attack, suggesting that sleep disturbances are not restricted to cluster periods (4). The disruption of the sleep–wake pattern can represent the first manifestation of an incipient bout: patients report nocturnal awakenings, trouble falling asleep, or restless sleep in the days preceding the onset of attacks (5). Brain areas participating in sleep regulation, circadian rhythmicity, and pain modulation are fully involved in the pathophysiology of CH. The pivotal role of the hypothalamus, revealed by functional brain neuroimaging, could explain the substantial sleep alteration in these patients, reinforcing the idea that sleep disorders are not mere consequences of the attacks (6). We describe the case of a patient affected by episodic CH who experienced a sleep disorder after starting therapy with verapamil.

## Case presentation

**Figure 1:**
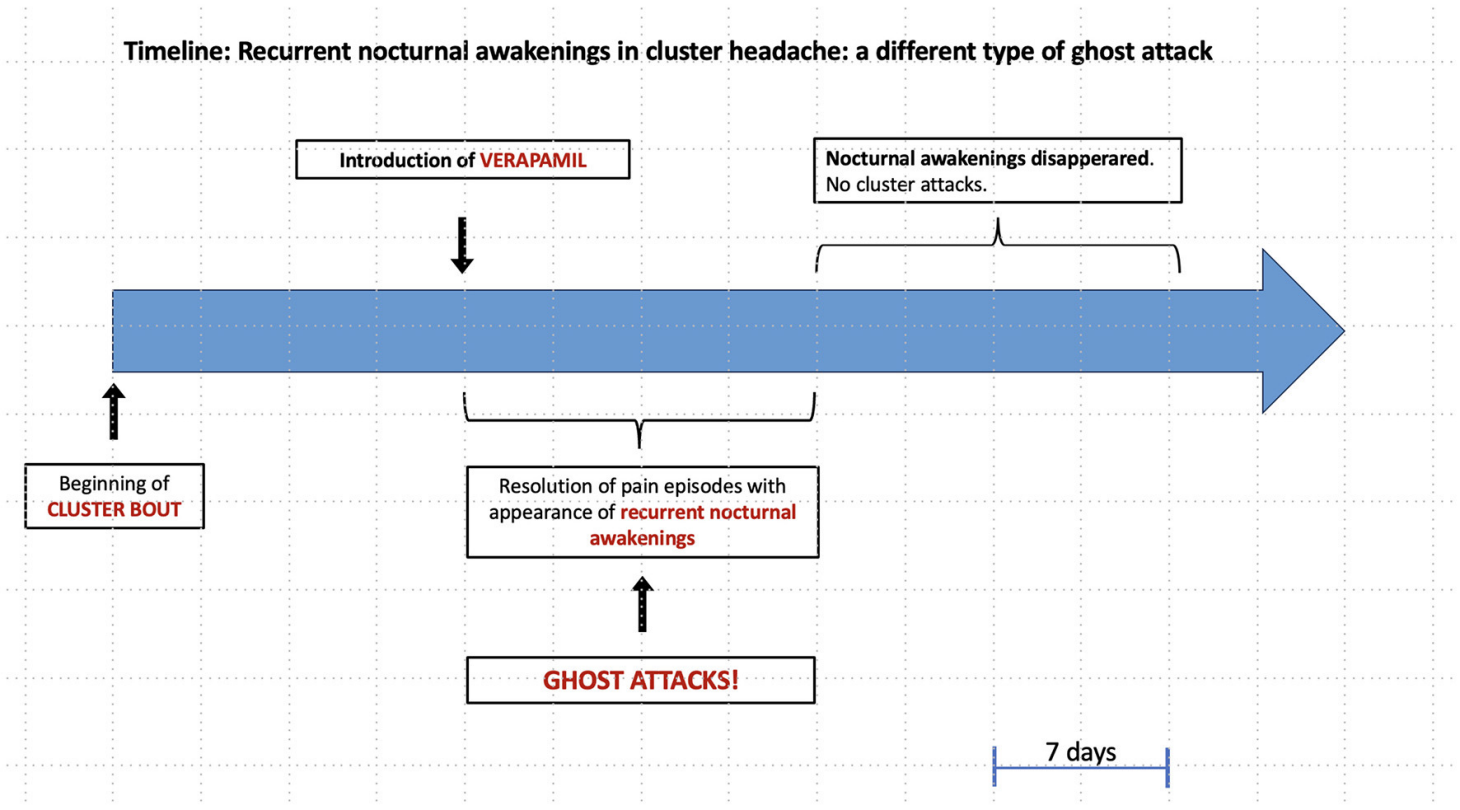
Timeline: Recurrent nocturnal awakenings in cluster headache: a different type of ghost attack.

Since 2019, a 47-year-old man has been affected by episodic CH [according to the International Classification of Headache Disorders, ICHD-3 criteria (7)]. He had only two bouts in his history. The first bout lasted for 15 days and resolved without treatment, while the latter was more severe and disabling. The attacks lasted for 120–180 min, and they were characterized by excruciating pain in the left orbital and temporal regions, associated with ipsilateral lacrimation, conjunctival injection, ptosis, eyelid edema, and rhinorrhea. Headaches occurred during the night, with one or two nocturnal attacks appearing at 11.30–12 p.m. and 4–4.30 a.m. A preventive treatment with verapamil was started, gradually increasing by 40 mg every 2 days until 120 mg daily. The patient had to monitor blood pressure and heart rate during therapy, which showed no significant changes. The follow-up visit was performed 2 weeks later: the patient reported only two other nocturnal attacks after the start of therapy. Interestingly, he started to experience consecutive nocturnal awakenings for a couple of weeks after attack resolution, without any pain or autonomic symptoms. These were described as sudden awakenings every night, always around the same time, around 4–4.30 a.m.; he was not agitated and did not need to get out of bed, but after the awakening, he had sleep disturbances characterized by vivid dreams (Figure 1).

The patient was otherwise healthy. General and neurological examinations, routine blood tests, electroencephalogram, and brain magnetic resonance imaging, including arterial and venous magnetic resonance angiography, were normal.

Regarding the sleep–wake habits, he did not complain of any sleep disorder and denied any need to take sleep medications in the past. This unusual phenomenon, characterized by a very regular pattern of presentation in the absence of a previous sleep disorder, has not been categorized as a specific sleep disorder.

## Discussion

To the best of our knowledge, this case represents the first description of recurrent cyclic nocturnal awakenings without pain and autonomic symptoms that occur in a patient with episodic CH during the active phase of cluster bout. In the literature, sleep disorders have been reported as the warning sign of an incipient cluster episode (8) even if the most common events of the pre-cluster period are the so-called “shadow attacks” (9, 10). In clinical practice, after the beginning of prophylactic therapy, it is of interest that patients often describe several symptoms resembling those of the pre-cluster period. Transient feelings of discomfort around the eye or mild pain in the typical localization of CH attack could be observed during the first days of treatment although the fast clinical improvement. These manifestations are facetiously called “niggles” by English sufferers or “ghost attacks” on patients' forums. In our case, the introduction of effective preventive treatment is followed by the appearance of cyclic nocturnal awakenings. The only description of a similar episode is from a woman affected by CH, who wrote on her blog “Have you ever woken up at 2 a.m. with the “rush” that a cluster attack is happening, but nothing happens? This is a ghost attack. I started experiencing them when I went on preventative medications (11).” According to her words, the nocturnal awakenings of our patient might be considered a different type of “ghost attack”.

In the literature, descriptions of ghost attacks or clear evolution of headache symptoms after the introduction of prophylactic therapies are lacking. Only Meyer and Hardenberg reported that in four patients affected by chronic CH, the presence of “tightness” was noted around the affected eye (without pain development) during therapy with verapamil (12). This drug is considered the first-line prophylactic treatment for CH although its exact mechanism of action is still unknown (13). It mainly acts by blocking L-type calcium channels, which are widely expressed in the central nervous system (14), modifying the release of neuropeptides, such as calcitonin gene-related peptide (CGRP), involved in the pain pathway. Therefore, by modulating the central activity of different neurotransmitters (15), verapamil can promote attack resolution. It seems to be more effective in episodic forms of the disease, and patients have the major advantage of taking the drug at appropriate times, according to their timetable of attacks (16). Indeed, the evening intake of verapamil can delay the onset of nocturnal attacks by ~1 h in episodic CH (17). The reduced efficacy in chronic CH could be explained by the decline of circadian features, with more irregular patterns of painful episodes. Different pharmacodynamic aspects of verapamil sustain a possible circadian effect. Calcium current seems to be partially controlled by the suprachiasmatic nucleus of the hypothalamus: The flux of calcium ions through the voltage-gated calcium channels changes in relation to the light-to-dark cycle, and L-type calcium channels participate in resetting the circadian rhythm during the late night (13). A recent animal study revealed how this drug could shorten the circadian period at both the molecular and behavioral levels in mice, eliciting sex-specific sleep changes (18). The effect of this drug on sleep architecture might be slower compared to that on pain pathways, necessitating a longer time of administration. The presence of “ghost attacks” in CH patients, even when represented by sleep disorders, should stimulate the continuation of therapy with verapamil, eventually increasing the dose, before switching to another medication. Furthermore, drugs such as lithium or melatonin, despite more pronounced effects on circadian rhythm, do not seem more effective in CH prophylaxis (19).

Cyclic nocturnal awakenings of our patient strengthen the importance of the hypothalamus in the pathophysiology of this primary headache. The orexinergic system seems to play a crucial role, both regulating sleep–wake cycle and modulating trigeminal nociceptive processing (20), but further studies are necessary to better define which neurotransmitters are mainly involved.

## Conclusion

Our case shows that nocturnal awakenings without any pain or vegetative symptoms may represent a sort of “ghost attack” in CH patients. In this light, sleep disturbances, similar to painful episodes, might be considered a manifestation of a still active cluster bout, thereby encountering the unmet needs of CH patients, as emerging from their blogs, potentially being a further element to evaluate the efficacy and duration of preventive therapy.

## Data availability statement

The original contributions presented in the study are included in the article/supplementary material, further inquiries can be directed to the corresponding author.

## Ethics statement

Written informed consent was obtained from the patient for publication of this case report.

## Author contributions

GG and VD made the diagnosis. GG was responsible for case management and wrote the first draft of the manuscript. MG and MA contributed to completing the information related to the case. MG participated in the revision process. VD edited and approved the final version of the manuscript. All the authors read and approved the final manuscript.
